# Supplementary material for: MetaRibo-Seq measures translation in microbiomes
Source: Nat Commun. 2020 Jun 29;11:3268. doi: 10.1038/s41467-020-17081-z (PMC7324362; doi:10.1038/s41467-020-17081-z)
Supplement: Supplementary file 10 — Supplementary Data 7 [file 41467_2020_17081_MOESM10_ESM.zip › File2/Confidence_High_Taxonomy/142009_out.krona.html]

Javascript must be enabled to view this page.

members
magnitude
magnitudeUnassigned
count
unassigned
taxon
rank

142009\_out

2

superkingdom
2
2

2
1224
phylum

28216
class
2

2
order
80840

2
family
119060


SRS043841\_contig\_number\_contig-100\_4027.49769SRS044535\_contig\_number\_contig-100\_12907.57297
2
48736
genus
